# Patient perspectives on an intervention after suicide attempt: The need for patient centred and individualized care

**DOI:** 10.1371/journal.pone.0247393

**Published:** 2021-02-19

**Authors:** Laurent Michaud, Yves Dorogi, Sophie Gilbert, Céline Bourquin

**Affiliations:** 1 Psychiatric Liaison Service, Lausanne University Hospital, Lausanne, Switzerland; 2 Université du Québec à Montréal, Montréal, Canada; University of Toronto, CANADA

## Abstract

**Background:**

Many types of intervention exist for suicide attempters, but they tend not to sufficiently consider patient’s views.

**Aim:**

To identify useful components of a previously evaluated intervention after a suicide attempt from the patient’s views and to better understand the process of recovery.

**Method:**

Forty-one interviews with suicide attempters were qualitatively analysed. Views of participants (i) on the components of the intervention (nurse case-management, joint crisis plan, meetings with relatives/network and follow-up calls) and (ii) their recovery were explored. The material was analysed by means of thematic analysis with a deductive-inductive approach.

**Results:**

Participants valued the human and professional qualities of the nurse case-manager, and appreciated follow-up calls and meetings. However, their views diverged regarding for instance frequency of phone calls, or disclosing information or lack thereof. Interpersonal relationship, suicide attempters’ own resources and life changes emerged as core recovery factors.

**Discussion:**

The study highlights the figure of an engaged clinician, with both professional and human commitment, aware that some suicide attempters put more emphasis on their own resources than on delivered health care.

**Conclusions:**

Interventions should consider the clinician as the cornerstone of the tailored care valued by suicide attempters.

## Introduction

Suicide attempts are a major public health issue and the most important risk factor to ultimately bring it to completion [1]. It may create stigmatization [2], difficulties with help-seeking [3] and also represents an important economic burden [4]. A large range of interventions have been developed and evaluated to care for people who have attempted suicide, ranging from minimal follow-ups (e.g. postcards or texting) to long-lasting psychotherapeutic interventions [5]. The obtained outcomes specifically concerning a decrease in repetition of suicide attempts or death by suicide are heterogeneous [5,6]. This may be explained, among other things, by the different level of acceptance of the intervention and how they are tailored to suit the patient’s needs [7]. Over all, the perspective of the service users on the provided care after a suicide attempt is still insufficiently known [8]. Indeed, qualitative research in suicidology tends to focus on the experience related to suicidal behavior [9], and we actually did not identify any study that explores the patient’s experience or views on a specific intervention.

In a previous study, we designed and tested an intervention for people admitted in our emergency department after attempting suicide [10]. The intervention was lead by a nurse case manager and supplemented the care as usual after a suicide attempt in our unit. Care as usual consists of either an immediate referral to a treating therapist, an outpatient crisis intervention in our unit or a psychiatric admission.

Expecting a cumulative effect, we included several components in the “add-on intervention”, issued from a literature review of best practices and based on our clinical experience. These components included coordination of care by a nurse case manager [11], the development of a joint crisis plan [12], early meeting with relatives and the already constituted care network [13] as well as phone contact over the span of three months after the suicide attempt [14]. We encountered important difficulties in implementing our intervention: a lack of interest among potential participants, and organizational challenge around the joint crisis plan and the early meetings [10]. These findings are in line with the following observations: suicide attempters are relunctant to engage in treatment [15], there is a discrepency between their needs and the care provided and a limited communication with the clinical staff [7].

Considering these findings, the aim of the present study was to explore views of patients who attempted suicide, on our intervention and on their recovery process. It should also shed light on potential ways to impact the experience of these patients, enhance their care and better adapt a future intervention to their specific needs.

## Method

### Ethics statement

The local ethics committee (CER-VD) approved the study (Protocol 411/13). A verbal informed consent was obtained from every participants and audio-taped at the beginning of every interview (see participants section for further details).

### Intervention

Our intervention following suicide attempt is exhaustively described in a previous publication [10]. Designed as an add-on to care as usual (CAU) for patients admitted for a suicide attempt, it included the following four core components: case-management by a mental health nurse, joint crisis plan, early meetings and follow-up calls (see above).

### Participants

Of the 107 suicide attempters for whom a psychiatric consultation was requested during the period of the above mentioned study, 22 were excluded for the present study (18 prisoners; 1 member of the Department staff; 1 deceased person; 1 person with no contact available), while 85 were contacted by mail and then by phone. Thirty-three could not be reached and 11 declined participation. The investigation was thus based on phone interviews with a sample made up of the 41 patients who consented to participate in the study. They belonged to different groups: patients who (i) were included in the aforementioned study and participated in the intervention, in addition to care as usual (INT group; n = 13), (ii) refused to participate in the study and received care as usual (n = 5), and (iii) were not included in the study for logistical reasons, and also received care as usual (n = 23). We have decided to include patients who did not take part in the intervention [(ii), and (iii)], because they can potentially offer alternative perspectives on the needs of those specific patients. They form the CAU (care as usual) group in this study. The patients were contacted, informed of the study and interviewed by one of the authors (YD, a psychiatric nurse, experienced in giving care to suicide attempters) after obtaining informed consent. The interviews were conducted 12 to 16 months after the intervention. Telephone interviews were preferred to face-to-face interviews based on the assumption that the participation rate would be higher if participants did not have to attend the hospital for a research appointment.

### Data collection

The interviews were structured to cover key themes (components of the intervention) but also included emerging issues. The interview guide focused on the respondents’view regarding the intervention: 1) based on their own experience (INT group, participants with experience-based responses) or 2) based on its description by the investigator (CAU group, participants with description-based responses). In this last case, the investigator described each component in detail before asking participants for their opinions. For participants in the INT group, the investigator went over the intervention offered. In addition, individual recovery factors were explored, i.e. factors helping participants after their suicide attempt, in both groups. We indeed assumed that views on the intervention were influenced by their experience of recovery, be it related to care or not. The phone interviews were recorded. They ranged from 6 to 30 minutes, with an average of 13 minutes.

### Data analysis

The interview material was analyzed by means of thematic analysis with a deductive-inductive approach [16]. A coding frame was developed from iterative listening to interviews by three investigators–a psychiatric nurse (YD), a psychiatrist (LM), a social scientist (CB)–and referring to the questions of the interview guide. To determine reliability of the coding frame, about 10% of the interviews were coded independently by the investigators who agreed on 80% of the themes. Disagreements in coding were then resolved through discussions until a consensus was reached. They consistently discussed the content and definitions of the most salient themes and highlighted the links (opposition, hierarchy, etc.) between them. Data saturation was reached after 25 interviews, but all interviews (n = 41) were coded (convenience sample). Excerpts of the interviews were selected and transcribed to exemplify the findings. We did not use a qualitative software package to help with the analysis; given the various levels of experience with qualitative research of team members, we preferred low technology techniques [16]. This study followed the COREQ checklist criteria for reporting of qualitative research.

With respect to the investigators, LM and YD participated as senior clinicians in the development of the intervention but were not involved in the care of the participants; as a social scientist, CB offered a different non-clinical expertise. SG, a clinician psychologist experienced in qualitative research joined the team later, providing a supplementary perspective. Such interdisciplinarity enabled in depth and sound discussions of findings and interpretations.

## Results

### Views on the intervention

This section reports on the views (experience-based or description-based, see above) of patients in INT group and CAU group regarding the four core components of the intervention: case-management, joint crisis plan, early meetings and follow-up calls. Participants are identified by the letter P and a number when quoted. Table 1 summarizes the main themes.

**Table 1:** Themes.

**1a) Views on the overall intervention**	Neglect of social dimensions			
	Too much attention on the suicide attempt			
	Organizational issues: high number of involved health professionals			
**1b) Views on the components**	Case manager	Joint Crisis Plan	Meetings with family (F) and networks (N)	Follow-up phone calls
	Interaction with the nurse case manager • Being listened as individuals • Possibility of dialogue • Therapeutic bond Openness of the case manager regarding their experience • Benevolence and attentive listening • Attention paid to the psyche Joint reflection on the suicide attempt • Explore the reasons • Provide meaning • Face consequences • Welcome support Trustful relationship • Ensure continuity of the follow-up Lack of real concern • Unfriendly and uncaring case-manager	Bad timing • Insufficient affective distance from the act • Emotional and logistic burden • Understanding difficulties Risk of redundancy Need of prior relationship	Source of support (F) • Providing a sense of help • Being listened and understood Opportunity to provide explanations and to discuss (F) • Making room for the family perspective and involvement • Place to explain the suicidal crisis to the relatives • Place where involvement of the relatives in the events can be demonstrated Sharing of information (N) • Transmission of information • Presence of a mental health professional who can provide information Intrusion (F) Organizational issues (F) • Choice of participants • Timing (too soon) Confronting several health professionals (N)	Openness toward suicidal patients • Opportunity to talk and to express feelings • Attention paid to suicidal thoughts • Non-judgmental support • Fostering of a trustful bond Convenience • no travel needed Emergence of a reflexive process Alternative to hospitalization Lack of planning and shared scheduling • Risk of intrusion (too frequent) or the contrary (not long enough) Depersonalized contacts Risk of overcare Organizational aspects
**2) Recovery factors**	Interpersonal relationships • Family, friends, colleagues • Health professionals from care as usual • Other health professionals and especially general practitioners	Own psychological resources • Self-reflection, introspection, guilt towards others • Faith	Life changes • New hobby (e.g. painting, acting) • Professional activity • Disengagement from conflicting relationships	

#### The case manager

In both groups, most of the participants expressed positive comments on the case management component. They especially valued the **interaction with the nurse case manager** (bold characters indicate main themes). Participants of the INT group felt that they were *listened to as individuals* (italic characters indicate subthemes), that *a dialogue took place*, and that the *therapeutic bond* with the case manager was important. This last point was also endorsed by participants of the CAU group.

Participants in INT group also appreciated the **openness of the case manager regarding their experience**: *his/her benevolence and attentive listening*, or the *attention paid to the psyche* (and not only to the body).

*I really liked the person who was taking care of me; she was really empathetic and it was not just professional*, *she really had something generous in her*. ^P5^

Moreover, the possibility **to reflect together** with the case manager **on the suicide attempt** was perceived by both groups as an opportunity *to explore the reasons for the crisis*, *to provide meaning to the suicidal attempt*, and even *to face its consequences*. These discussions may as well be seen as a *welcome support “to know where we stand after this event”* ^P8^

The case manager may finally *ensure the continuity of the follow-up* and consequently **foster a trustful relationship**.

*Truly said*, *when I’m in the middle of a crisis*, *I like to be in contact with the same person if I had a good contact with him/her*. *Knowing that I can contact a specific person*, *it helps me* ^P1^

One INT group participant stressed, however, the **lack of real concern** of the case manager: he felt the case manager was *unfriendly and uncaring*.

#### The joint crisis plan

In contrast with the previous component, the perceptions of a joint crisis plan intended to avoid future suicidal crisis were unfavorable in both groups. The criticisms mostly addressed the **bad timing**: since the length of stay in the emergency ward is limited, *the affective distance from the suicidal act* was considered *insufficient*, and the joint crisis plan was viewed as an *additional emotional and logistic burden*.

*I think it is good to have it [the joint crisis plan]*, *but not on the day we are in the hospital*. *When I get admitted*, *I’m aggressive*, *the questions piss me off*, *I don’t feel like talking*. *I’m feeling better on the day after and I feel more like talking*, *saying something* ^P10^

In addition, several participants emphasized that patients are acutely affected physically and mentally, thus having *difficulties to understand what is at stake with the joint crisis plan*.

*I’m a bit divided I must say*, *to establish this kind of thing directly following admission*, *it’s clear that it was not possible regarding the state of mind I was in* ^P11^

Futhermore, in both groups, some participants considered that **having a prior trustful relationship with the case manager** is a necessary condition to elaborate a joint crisis plan and that a **risk of redundancy with previous work done with their therapist** exists. Writing strategies such as a joint crisis plan may nevertheless be a *resource to prevent another suicide attempt acting out* according to a few participants of the INT group.

#### Meetings with family and networks

Family meetings, the third component of the add-on intervention, were positively evaluated by both groups of participants. They saw these meetings as a potential or effective **source of support** for themselves or their significant others, *providing a sense of help* and the *feeling of having been listened to and understood*.

*Doing this meeting with my mum […]*, *it showed that I often have a mask in front of everyone and no one sees how bad I feel*, *it made them realize that I didn’t feel good* ^P15^

Having or not experienced these meetings, several participants identified an **opportunity to provide explanations and to discuss** with physicians acting as mediators. They deemed that meetings could represent a way *to make room for the family perspective and involvement*. Those meetings could also represent *the place where the suicidal crisis can be explained to the relatives* or where *their involvement in the events can be demonstrated*.

*I find it most important*, *the family members need to be heard by professionals*, *because they are suffering and facing a huge shock […] that the professionals can also explain to the family the distress of someone attempting suicide and what can be done in the future* ^P20^

Besides, some participants highlighted a number of negative aspects of the family meetings whether they experienced them or not: e.g., regarding organizational issues such as the *choice of who participates in the meeting* and the *timing* (too soon for some participants, too late for others), and the **apprehension of being judged by the relatives.**

Facing family members has also been considered as an **intrusion**, for instance when potentially hurtful information for relatives have to be disclosed.

Since only one participant in the INT group experienced the network meeting (due to reluctance of most patients and/or organizational issues) [10], the responses mostly relied on the description provided by the investigator. The **sharing of information** was particularly valued by the participants. They stressed the importance of the *transmission of information* and the advantages related to the *presence of a mental health professional who can provide information based* on the continuity of care.

**Confronting several health professionals** was negatively perceived by some participants who described themselves as solitary persons, reluctant to the idea of involving other people in their care, and uncomfortable with a group interaction. Also, one participant considered network meeting as overcare.

#### Follow-up phone calls

Regular phone calls during the weeks following the suicide attempt were the long-term part of the add-on intervention. Both groups endorsed those calls which were seen as reflecting **openness toward suicidal patients**. Many participants of both groups valued the *opportunity provided to talk* and *to express feelings*, *the attention paid to suicidal thoughts*, and *the non-judgemental support provided*. They felt that the calls *fostered a trustful bond* between the nurse case manager and the patient.

*I didn’t even feel like I was dealing with a shrink nurse*, *I felt like I had a friend calling back to check in on me* ^P4^

This specific **modality** of intervention was also commented. Some participants appreciated that *no travel was required* and the *consistency*.

Futhermore, several participants in both groups outlined *the temporal and relational continuity* induced by the phone calls and considered that they allowed the **emergence of a reflexive process** while representing an **alternative to hospitalization**.

*This can really bring you something; it forces you to ask yourself some questions* ^P14^

However, both groups also expressed criticism on phone calls, for instance because of their **lack of planning and shared scheduling.** They *were experienced as intrusive* when *too frequent*, or on the contrary, some participants deplored the *limited duration of the said intervention* (three months).

*It is a necessity but the call shouldn’t be too frequent; there should be an agreement with the person on how many times a week and at what schedule* ^P21^

Some participants from both groups considered the calls as **depersonalized**, while for some others phone calls lead to **overcare**.

*My doctor was already calling me and watching how I was doing*, *so it [the phone calls included in the intervention] kind of oppressed me actually* ^P15^

Finally, some participants put forward several negative aspects, which did not concern one component but rather the whole intervention. The *neglect of social dimensions*, such as professional reintegration of suicide attempters was underlined. Moreover, some participants bemoaned that *too much attention was being paid to the suicide attempt*. Some of them argued that the suicide attempt could be considered as a “personal choice” which should be respected, as well as the reasons leading to this act. Others would mention that this difficult event had to be forgotten in order to avoid the shame.

*For me it wouldn’t have been ok because I just wanted to forgot what I did; I was ashamed so I didn’t want to be reminded all that much* ^P22^

**Organizational aspects** such as *the growing number of involved health professionals* due to the intervention–even more when patients were already treated and followed by a psychiatrist–were also criticized.

### Recovery factors

In order to gain insight into the experience of recovery during and beyond care as usual or the add-on intervention, the study also explored factors considered as helping by the participants, following their suicide attempt. Three main themes were identified, which echoed the results on the components of the intervention.

Participants valued **interpersonal relationships**, specifically the opportunity to be supported by, and to interact with, significant others; they alluded to their *family*, *friends and colleagues*.

*My family and friends realized that it was going too far*, *that something had to be done*, *that I wasn’t kidding*, *I was tired* ^P12^

The care as usual was considered beneficial, when participants felt understood, *could express themselves without being judged* and *felt supported and listened to*.

*I was really able to express myself*, *I felt understood in the suffering I was in*, *these persons [the staff] were highly supportive* ^P9^

Different community or support groups (e.g. church-related groups) were also mentioned by the participants. In addition, *health professionals*, *and especially general practitioners*, were appreciated, since they provided the opportunity to share personal problems that were not discussed with relatives.

Participants then also relied on their **own psychological resources**: *self-reflection*, *introspection*, *guilt towards others* as well as *faith* were reported as support for recovery.

*I’ve been thinking and*… *I have little kids who need me*, *and I can’t waste my time thinking about stupid things like that* ^P27^

Lastly, **life changes** were viewed by some participants as having played an important role in their recovery. Positive changes included involvement in *a new hobby* (e.g. painting, acting) or in a *professional activity* as well as *disengagement from conflicting relationships*.

## Discussion

Following the challenging implementation of an add-on intervention after suicide attempts [10], this study aimed to explore views of suicide attempters on this specific intervention and on their recovery process.

The diverging, and at times antagonist views of participants with regard to the components of our intervention seem to be related to how they accounted for (i) the way they understand their suicide attempt and how they can recover from it, as well as (ii) the organization of the components of the intervention.

Firstly, some participants deplored that too much attention was being paid to the suicide attempt, referring for instance to the fact that this act could be considered as a “personal choice”. Furthermore, differences between participants were observed with respect to how they may be helped and cared for. Some of them found phone calls too frequent, others bemoaned their termination; some wanted to share information with family, others did not. Some participants valued a solid and intensive relationship with the nurse case manager after their suicide attempt and liked to share their difficulties with relatives. Others considered their own resources as the most important factor, and family meeting or the presence of several mental health professionals as overcare. These aspects may reflect alternative views on suicide attempt, either “medical-related” (and thus requiring professional care) or “social-related” (and thus in need of other interventions). This last view on suicide attempt, which is more socially explained as “something that happens” may be in fact related to what other participants underlined, the *neglect of social dimensions*, such as professional rehabilitation of suicide attempters, and the fact that they highly valued their own resources in their recovery process. Altogether, this challenges the psychological perspective which considers suicidal attempts as pathological and medically explained [17]. In this regard, another study showed that suicide attempters criticized the fact that clinicians tend to focus on a medical perspective, forgetting to ask their patients “why” they attempted suicide [18]. This calls for an approach targeting the “how and why” of the suicide attempt rather than suicidal risk assessment [19], such as Attempted Suicide Short Intervention Program (ASSIP) [20].

From another point of view, this highlights that people attempting suicide may want to “cover” what happened as much as “uncovering and elaborating” around their suicidal attempt, as it would be usually proposed. This could be the expression of an ambivalence (“discovering/disclosure” versus “covering” hot topics), which should be considered when approaching suicide attempters. Indeed, a recent international study showed that a majority of persons not seeking help from suicidal issues perceived a low need for treatment or wanted to solve their problems by themselves [21]. In our hospital, the vast majority of patients did not have the choice of being admitted to the emergency ward: emergency assistance was called by close relatives. Psychiatric consultation was then offered very actively, following guidelines which strongly recommend psychiatric consultation after self-harm. This duality between “covering” and “discovering/disclosing” was also observed both with the family and with networks. While some participants in our study wanted to share information and emotions with their family, others perceived family meetings as an intrusion in their intimacy. This may mirror the way suicide attempters seek help or not. Clinicians must find a balance between respecting patients intimacy and vulnerability, and working toward elaboration on the context of the suicide attempt.

Secondly, regarding the organization of the components of intervention, our results show the importance of taking into account the “how” and the “when”. Appreciation of the components and feasibility of the implementation (framework of the intervention) appeared together as necessary conditions. For instance, the clinical concepts underlying the joint crisis plan were valued by patients but its implementation was strongly criticized: bad timing, administrative burden just after suicide attempt, etc. The same holds true for phone calls, with participants deploring the lack of planning and criticizing their quantity (too many or too few). Also, regarding the add-on intervention as a whole, the growing number of involved health professionals and the very timing of this intervention were deemed problematic.

Our results, by showing that people who attempt suicide do not share a common view on the intervention and how it is displayed, raise the question of how to adapt an intervention to be consistent with the patient’s needs. Multiple post-suicide attempt interventions have been studied [22] but they followed almost always a “one size fits all” approach, namely same intervention for everyone. As an exception, Vaiva et al. developed a “two-steps” intervention for first-attempter versus repeaters, with inconclusive results [23]. Our results seem to indicate, rather, that intervention needs are specific to individuals. Other studies point in this direction as they showed that adult people who self-harm value tailored care [24] or that young men clearly prefer the help of their peer network [25]. One way to adapt multicomponent intervention would be to offer the possibility for suicide attempters to select the components of their intervention, which would however be quite challenging to implement. Interventions based on a face-to-face specific relationship could be a better option (e.g. psychotherapy). In such a setting, the therapist has to adapt to each patient; he/she intuitively “choose” which intervention and techniques are appropriate for a specific patient. It relies mostly on the quality of the encounter, which echoes our results showing that interpersonal relationships are central: be it with regard to the nurse case manager, to meetings with family, to phone calls or when addressing what helped in recovery. Our results seem therefore to speak for such a “one-on-one” intervention. Indeed, such an approach could take into account how patients understand their suicide attempt and how they plan to recover from it, e.g. by avoiding to overcare when patient prefer to rely on other resources. Furthermore, it may allow tailoring the organization of the intervention, e.g. overcoming the above-mentioned bad timing of joint crisis plan by delaying its completion or adapting the frequency of phone calls to specific needs of the patient. Finally, it would improve the continuity of care, an issue frequently raised by participants.

### Limitations

Our sample of participants was heterogeneous with first-time attempters versus repeaters, people having taken part or not in the intervention, and the study design did not allow determining what characteristics these different populations share in common. While our initial study showed that first-time attempters were especially difficult to engage in the intervention [10], we were not able to show specific differences between this group of patients and other suicide attempters.

Furthermore, length of time between the end of the evaluated “interventions” (CAU and add-on intervention) and the phone interviews was quite long (12–16 months), given that (i) the study was designed in continuation to the results of the first study and (ii) the procedure to obtain ethical approval was a lengthy process.

## Conclusion

Rather than offering “one size fits all” interventions, our results show the need of patient-centered *and* tailored care in intervention after suicide attempt. In this regard, clinicians appear to be key, as professionals *and* as persons. Of course, they should offer professional and medical support, but they also have to be aware that some suicide attempters put more emphasis on their own resources and personal relationships than on delivered health care when it comes to recovery. But above all, they should be authentic, accessible, non judgmental, and therefore able to build trust and to be “carers” for those in pain. Therefore, a committed clinician appears the core component of the tailored care valued by patients who made a suicide attempt.
